# Metagenomic Tools as Predictive Approaches to Decipher Soil and Rhizosphere Microbial Communities and Their Functions for Sustainable Agriculture

**DOI:** 10.1155/ijog/3098615

**Published:** 2026-10-05

**Authors:** Oluwaseun Emmanuel Shittu, Jing Ge, Olubukola Oluranti Babalola

**Affiliations:** ^1^ Soil-Plant-Microbe Interactions Group Faculty of Natural and Agricultural Science North-West University Mmabatho South Africa nwu.ac.za; ^2^ Institute of Food Safety and Nutrition Jiangsu Academy of Agricultural Sciences Nanjing China jaas.ac.cn; ^3^ Department of Life Sciences Imperial College London Silwood Park Campus Ascot Berkshire UK imperial.ac.uk

**Keywords:** agricultural sustainability, amplicon sequencing, beneficial microbiome, functional genes, shotgun sequencing

## Abstract

The increasing demand for resilient and sustainable food production necessitates a comprehensive understanding of rhizosphere microbial communities and their functional roles in enhancing plant health, nutrient acquisition, stress resilience, and crop productivity, thereby supporting food security and sustainable agriculture. However, soil microbiomes are composed of complex and unculturable communities of beneficial microorganisms that drive critical ecosystem functions, which traditional culture methods cannot capture. This limits the understanding of their roles and impacts on plant health and agricultural sustainability. Metagenomic tools have emerged as transformative approaches for characterizing microbial diversity in soil. Therefore, this review presents an overview of how metagenomic techniques can be harnessed to predict microbial community structures and their associated functional potentials, ultimately contributing to the development of resilient farming strategies. Next‐generation sequencing and bioinformatics enable metagenomics to analyze microbial genetic material in soil and rhizospheres. This study provides insights into microbial diversity and profiles of key functional genes linked to nutrient availability and soil functions. We discuss the use of metagenomics tools to predict soil health and microbial functions for sustainable agroecosystems, emphasizing predictive models that help farmers optimize yields and minimize environmental impact. This review explores how metagenomics tools in agriculture can serve as a predictive model to transform our understanding of soil–microbe interactions. This advancement can facilitate the formulation of novel, more resilient, productive, and sustainable agricultural systems.

## 1. Introduction

The soil and rhizosphere microbiomes are increasingly acknowledged as fundamental components for plant health, nutrient cycling, and resilient agriculture [1]. The rhizosphere is often referred to as the confined soil compartment intimately shaped by rhizodeposits and related microbes. It supports a multifaceted and beneficial consortium of microbes, comprising virus bacteria, archaea, and fungi [2]. These microbial populations engage in diverse interactions with plants, facilitating growth, augmenting stress tolerance, and nutrient mediation, which are vital for plant development [3]. Lidbury et al. [4] demonstrated that these beneficial rhizosphere microbiomes are capable of engaging in diverse functions, including solubilization of phosphate and transformation of atmospheric nitrogen (AN) into usable forms, as well as plant growth hormone synthesis. Similarly, they also help suppress harmful soil organisms by competing with and secreting antimicrobial substances [5].

However, microbial community diversity and functions are integral to plant health and wider ecosystem functions, including the maintenance of soil architecture, breakdown of organic material, and cycling of nutrients [6]. Previous findings have shown that the makeup of the rhizosphere microbiome is influenced by the plant′s genotype, the type of soil, and the prevailing environmental conditions [7, 8]. Nevertheless, certain functional traits, such as nitrogen (N) fixation and disease suppression, are frequently conserved across diverse systems. This functional redundancy is critical for ensuring the persistence of essential ecosystem processes, even as the soil community composition undergoes alterations in response to environmental changes such as fertilization amendments [9].

Despite their critical importance, conventional microbiological methods such as culture‐dependent techniques have encountered challenges in comprehensively characterizing the diversity and functions of soil and rhizosphere microbiomes [10]. Culture‐dependent techniques, which necessitate the in vitro proliferation of microorganisms, typically retrieve less than 2.5% of the entire community of microorganisms present in soil ecosystems [11]. Numerous soil microorganisms demonstrate resistance to cultivation, and those that are amenable to growth under laboratory conditions may not necessarily represent the most functionally pivotal constituents of the community of microbes [12]. This constraint has historically impeded efforts to elucidate the full spectrum of interactions among microorganisms and their ability to proliferate for the production and well‐being of plants [13].

The advent of high‐throughput sequencing techniques such as shotgun metagenomics has greatly transformed the investigation of soil and rhizosphere microbiomes. Metagenomic methodologies, such as shotgun sequencing, enable scientists to examine the entire gene pool of microbiome populations from environmental samples without cultivation, thereby eliminating the requirement for cultivation [14]. This approach has facilitated the characterization of previously uncharacterized microbiome taxa and the clarification of their functional diversities related to the cycling of nutrients, resistance to disease, and facilitation of plant development [15]. It has also helped in profiling the microbial metabolic pathways linked to plant growth and development, thus providing novel targets for microbiome engineering and sustainable agricultural practices [16].

Furthermore, Next‐Generation Sequencing (NGS) has facilitated the advancement of predictive modeling and the strategic manipulation of microbiomes. By integrating metagenomic datasets with network analysis and machine learning models, scientists are able to identify critical microbial taxa as well as functions that underpin beneficial plant–microbe interactions [17]. This advancement has facilitated the development of bioinoculants and soil amendments, which are designed to recruit or augment beneficial microbial communities, thereby increasing crop productivity and resilience while diminishing the reliance on chemical inputs [18]. In extreme environments, such as arid agricultural systems, metagenomic investigations have revealed a high degree of microbial population and functional diversity [19].

In relation to Sustainable Development Goal (SDG) 2 (Zero Hunger), the application of metagenomics has significantly augmented food production by employing microbial technologies aimed at enhancing agricultural productivity, soil health, and the sustainability of farming practices. This approach facilitates the identification of beneficial microorganisms that can be utilized in the formulation of biofertilizers and biopesticides, thereby contributing to increased crop yields while concurrently reducing the dependence on harmful chemical agents [13]. In a similar vein, metagenomics constitutes a crucial component in the pursuit of SDG 13, specifically concerning climate change research, by facilitating comprehensive environmental monitoring. This capability allows for the comprehensive collection of data, which is instrumental in the development of adaptive strategies designed to mitigate the impacts of climate change and environmental monitoring and increase ecosystem resilience [20].

Therefore, this review explores the use of metagenomics to profile soil and rhizosphere microbial communities and their roles in sustainable agriculture. This study highlights modern techniques for predicting microbial composition, diversity, and functions, emphasizing their benefits for plant health, nutrient cycling, resistance to diseases, and stress tolerance. This review discusses the integration of metagenomic data with traditional farming and future research, including the use of machine learning, to improve sustainable agriculture and soil health.

## 2. Rhizosphere Microbial Communities: Composition, Diversity, and Functional Dynamics

### 2.1. Microbial Diversity and Community Composition in the Rhizosphere

The microbial communities in the rhizosphere are among the most complex and strong predictors of crop productivity in soil environments, serving critical functions in terms of plant health, nutrient cycling, and agroecosystem sustainability [21]. Root exudates such as sugars, amino acids, and organic acids as well as associated microorganisms (bacteria, fungi, and archaea) in the rhizosphere have a direct effect on the rhizosphere, acting as focal points for microbial activities and functions [22]. This phenomenon is distinguished by intense interactions between the roots of plants, mineral nutrients such as N input and various groups of beneficial microbes inhabiting the rhizosphere of plants, each contributing distinctly to soil functionality and plant performance [23].

The structure of microbial communities within the rhizosphere is further influenced by a combination of crop species, soil properties, agricultural management practices, and ecological factors [24, 25]. Typically, bacteria represent the most numerous and dominant group, with phyla including Firmicutes, Proteobacteria, Bacteroidetes, and Actinobacteria (Table 1) frequently reported across different crops and environments [41]. Fungi, including arbuscular mycorrhizal fungi (AMF), such as *Claroideoglomus claroideum*, *Claroideoglomus etunicatum*, *Claroideoglomus luteum*, and *Rhizophagus irregularis*, function as essential mediators of nutrient uptake and crop resilience [42], whereas other fungal groups, such as *Trichoderma afroharzianum* and *Trichoderma longibrachiatum*, contribute to the decomposition of organic substances as well as disease suppression [43]. Archaea, although less diverse, are important for N cycling, particularly in ammonia oxidation, and their abundance is influenced by soil pH and nutrient status [44]. However, viruses, although less studied, have been shown to modulate microbial population dynamics and facilitate dynamic activity patterns that correlate with distinct host niche differentiation and metabolic strategies, indirectly influencing plant health and soil function [45].

**Table 1:** Key genes and functional markers of the soil and rhizosphere microbiomes for predicting agricultural sustainability.

Key genes/functional markers	Pathway(s) or metabolic processes	Functions in agricultural sustainability	Taxa involved	Metagenomics techniques used	Source of soil used	References
i. Phosphate (P) solubilization genes (*PQQGDH* and *ugpBCQ*)	Phosphate solubilization and transport	Increases phosphate bioavailability, supports sustainable P management in agroecosystems	Metagenome‐assembled genomes (MAGs) affiliated with Cyclobacteriaceae and Vicinamibacterales	Genome‐resolved metagenomics and amplicon sequencing	Wheat–maize rotation system	[26]
ii. Proline metabolism genes, 2AP precursor biosynthesis genes	Proline metabolism, 2AP aroma biosynthesis	Enhance rice aroma/yield via bioinoculants; sustainable cultivation of low‐yield scented varieties	Proteobacteria and Actinobacteria	Shotgun metagenomics	Scented rice rhizosphere	[27]
iii. N‐fixation (*nifH*), siderophore (*pchB* and *entF*), HCN biosynthesis (*hcnB*), ACC deaminase (*acdS*), phenazine (*phzF*), P‐solubilization (*appA*), butanediol (*butB*), methanol utilization (*xoxF*), acetoin (*budC*), N‐metabolism (*glnA, glnB*, *gltB*, *gltD*, and *nirB*), and IAA biosynthesis (*ipdC* and *iaaM*).	Butanediol/acetoin biosynthesis, nitrogen fixation, ACC deaminase, phosphate solubilization, phenazine, siderophore, HCN, stress resistance (oxidative, glutathione, catalase, peroxidase)	Plant growth promotion, nutrient cycling, stress mitigation, biocontrol	Firmicutes, Bacteroidetes, Actinobacteria, Proteobacteria, Acidobacteria, Chloroflexi, Verrucomicrobia, Tenericutes, Planctomycetes, Cyanobacteria, and Chlorobi (endophytic microbiome)	Shotgun metagenomics	Maize root (with organic, inorganic, and no fertilizer treatment)	[28]
iv. *chrA*, *arsB* (heavy metal resistance genes), heavy metal transporter family (P‐atPase, MIT, CDF, and ABC), N/P cycling genes.	Replication, recombination and repair, Inorganic ion transport and metabolism, Signal transduction mechanisms and Defense mechanisms were enhanced.	Phytoremediation of Cr‐contaminated soils, plant growth under metal stress, reduced chemical inputs	Proteobacteria, Actinomycetes, and Chloroflexi, Bacteroidetes, and Acidobacteria	Shotgun metagenomics	*Iris tectorum* rhizosphere under Cr‐stress	[29]
v. *gdh2* (ammonification), *narB* and *nasB* (denitrification), *narI*, *narJ* and *narH* (dissimilatory N reduction), nitrification (*amoA_ABC*) and denitrification (*narG, nirK, nirS, norB* and *nosZ*), *gltB* and *gltD* (glutamate synthesis), *gudB* and *gdhA* (ammonification), *nasA* and *nirA* (assimilatory N reduction), and *napA, napC, nirB, nirD* and *nrfA* (dissimilatory N reduction).	Nitrification, denitrification, ammonification, dissimilatory N reduction	Nutrient cycling and plant growth.	Proteobacteria, Actinobacteria, Chloroflexi, Nitrospirae, *Actinomadura*, Nitrospira	Shotgun metagenomics	Tea (*camellia sinensis*) soil	[30]
vi. Microbial genes encoding glycoside hydrolases (*GHs*), glycosyltransferases (*GTs*), carbohydrate esterases (*CEs*), auxiliary activities (*AAs*), carbohydrate‐binding modules (CBMs), and polysaccharide lyases (*PLs*)	Carbohydrate metabolism	It ccelerates soil organic carbon turnover; risks C loss, aids nutrient release and optimize N for C sequestration	Acidobacteria, Proteobacteria, Actinobacteria, Chloroflexi, and Bacteroidetes	Shotgun metagenomics	Tea (Camellia sinensis L.) plantation soil under long‐term fertilization	[31]
vii. Nitrogen fixation (*nrf* and *nirA*)	N, P, metabolism	Enhanced nutrient cycling, and plant growth promotion.	*Sphingomonas*, *Bradyrhizobium*, *Candidatus_Sulfopaludibacter*, *Phenylobacterium*, *Candidatus_Angelobacter* and *Humisphaera Sphingomonas*, *and Occallatibacter*	Whole Genome shotgun metagenomics	Maize–peanut intercropping	[32]
viii. *amoA*, *amoB*, *amoC and hao*, *nxrA*, *narI*, *napA*, *napB*, *nirK*, *nirS*, *norB*, *norC*, and *nxrB* genes	Nitrogen metabolism	Promotes soil N cycling, but excessive activity linked to reduced yield	Alphaproteobacteria, Caulobacteraceae, Bradyrhizobiaceae, Sphingomonadaceae, Phyllobacteriaceae, Mesorhizobium, Novosphingobium, Bradyrhizobium, Phenylobacterium and Sphingomonas	Shotgun metagenomics	*Panax* ginseng rhizosphere (nitrogen fertilization treatments at different dose)	[23]
ix. Carbon degradation, Nutrients cycling (N, C, P, S), N, C, P, S degradation pathways	N, C, P, S metabolism	Promotion of nutrients cycling and soil fertility.	Nutrients cycling and degradation microbes	Shotgun metagenomics (Quantitative)	Rice root (chemical fertilizer with or without biochar)	[33]
x. *amoA*, *amoB, amoC*, *nirK*, *cobQ*, *cobC*, *cobD*, *cobY*, *cobS*, *cbiC*, *cbiD*, *cbiE*, *cbiF*, *cbiG, cbiH*, *cbiL*, and *cbiT* genes	Carbon fixation and nitrogen metabolism	They support nutrients cycling such as nitrogen cycling and carbon fixation.	*Nitrosotalea* and *Nitrososphaera* (archaea)	Shotgun metagenomic sequencing	Agricultural upland soils	[34]
xi. *nirK*, *amoC*, and *ureC* genes	N metabolism	They support the cycling of nutrients including N fixation	Thaumarchaeota/Nitrososphaeraceae (archaea)	Shotgun metagenomic sequencing	A wheat–maize rotation system under different fertilization regime	[35]
xii. Archaeal enzyme systems	N, sulfur, and C metabolism	Nutrients cycling	Thaumarchaeota and Euryarchaeota (archaea)	Metagenomic whole‐genome shotgun sequencing	Moringa oleifera rhizosphere	[36]
xiii. AMF colonization markers	P metabolism	For plant resilience and nutrients acquisition	*Rhizophagus clarus*, *Rhizophagus irregularis*, and *Funneliformis geosporum* (AMF)	Metagenomic whole‐genome shotgun sequencing	*Maerua crassifolia* soil rhizosphere	[15]
xiv. *phnA, ppx, phnG, phnN and ugpQ phnF, phnH* and *phnL* (P mineralization)	P metabolism	Nutrient acquisition	Planctomycetes, Ascomycota and Basidiomycota, and the relative abundance of Mucoromycota, Blastocladiomycota (fungi)	Shotgun metagenomics sequencing	Chinese fire plantation	[37]
xv. *cysFUWAND*, *sat*, *narH*, *napAB*, and *nrfAH*, *nirK/S*, *norBC*, *cysH*, *cysJ*, and *sir*	Carbon, S, N, purine, pyrimidine metabolism	Nutrient acquisition	Ascomycota, Basidiomycota, and Chytridiomycota (fungi)	Shotgun metagenomics sequencing	Sugarcane rhizosphere	[38]
xvi. *amoA*, *amoB*, *amoC* and *hao genes*, *nifDH*, *narI*, *napA*, *napB*, *nirK*, *nirS*, *norB*, *norC*, and *nosZ* genes	Nitrogen metabolism	Nutrient acquisition	Ascomycota, Basidiomycota, and Mucoromycota (fungi)	Shotgun metagenomics sequencing	*Panax ginseng* rhizosphere	[23]
xvii. Genes and enzymes involved in the metabolism of carbon, nitrogen and defense mechanism	Carbohydrates and amino acids metabolisms	Nutrient acquisition and plant defense mechanism	Ascomycota, Basidiomycota, Mortierellomycota and Mucoromycota (fungi)	Shotgun metagenomics sequencing	*Salvia miltiorrhiza* rhizosphere	[39, 40]

Within these taxonomic groups are those that perform functions relevant to plant growth, including bacteria capable of N fixation, such as those belonging to the genera *Bradyrhizobium* and *Azospirillum*, which transform AN into an absorbable state [46], and those that solubilize phosphate, such as *Pseudomonas* and *Sphingomonas*, improving the availability of phosphate [47]. Rhizobacteria that increase plant growth (PGPR), including *Bacillus* and *Enterobacteria* genera, not only facilitate the acquisition of nutrients but also produce phytohormones, suppress pathogens, and improve the tolerance of plants to environmental stress [48]. The richness and abundance of these beneficial microbes are critical for maintaining soil health and supporting sustainable crop production.

### 2.2. Functional Roles of Rhizosphere Microbiomes

Microbial communities are central to the transformation of essential mineral elements, including N, phosphorus (P), potassium (K), and siderophore production in soil (Table 1). Furthermore, bacteria and fungi are involved in organic matter decomposition, liberating carbon and other minerals into the soil [49]. AN is converted by diazotrophic bacteria into forms that are absorbable by plants [50], whereas phosphate‐solubilizing microbes, including *Aspergillus flavus*, increase the bioavailability of P to enhance plant uptake [51]. Sulfur‐oxidizing and Sulfur‐reducing microbes facilitate sulfur cycling, which is essential for plant nutrition and soil health [52].

Similarly, plants and microbes in the rhizosphere are involved in a range of diverse interactions that can be beneficial (e.g., mutualism or positive), detrimental (e.g., antagonism or negative), and/or largely neutral with respect to plant fitness [53]. Mutualistic relationships, as observed between plants and AMF and PGPR, augment the uptake of nutrients, stimulate plant growth, and increase resistance to drought [54]. Antagonistic microbes, including biocontrol agents such as *Trichoderma* spp., suppress soil‐borne diseases and shield crops from harmful organisms. These interactions are facilitated by root exudates, which play a significant role in modulating plant health [55]. These interactions are facilitated by root exudates, which play a significant role in modulating the rhizospheric microbial community and, consequently, plant health [56].

Moreover, rhizosphere microbes perform a vital function in increasing the tolerance of plants to both abiotic and biotic stresses. Coinoculation with beneficial bacteria and fungi (such as *Rhizobium* sp., *Bradyrhizobium* sp., and *C. claroideum*) helps plants endure biotic and abiotic attacks by producing and/or modulating plant hormone levels, producing protective compounds, such as antioxidants, and improving nutrient acquisition [57]. Ratnaningsih et al. [58] further established that PGPR, including *Pseudomonas* spp. and *Bacillus* spp., produce ACC deaminase, which decreases the level of plants and helps plants survive drought stress. Similarly, AMF improve water and nutrient uptake, further supporting plant resilience under adverse conditions [59].

Similarly, archaea also play specialized roles in the rhizosphere, with distinct phyla driving key processes at the root–soil interface. Within this narrow zone, Thaumarchaeota are the main drivers of nitrogen cycling and use specialized ammonia monooxygenase enzymes to dominate the rate‐limiting ammonia‐oxidation step of nitrification by ammonia‐oxidizing archaea (AOA) [44, 60]. Concurrently, methanogenic lineages belonging to the Euryarchaeota phylum govern carbon dynamics, tightly regulating methane production and flux in anoxic environments such as iron‐rich paddy soil [61]. In addition to these biogeochemical cycles, both phyla actively support plant fitness by synthesizing growth‐promoting phytohormones such as auxins [62] and producing antioxidant enzymes such as catalase‐peroxidase to alleviate host oxidative stress under harsh environmental conditions [36]. Ultimately, the metabolic versatility of Thaumarchaeota and Euryarchaeota stabilizes the broader soil microbiome network, reinforcing biological resistance against pathogens and increasing crop resilience [63, 64].

Metagenomic profiling revealed several major functional categories with putative relevance to rhizosphere processes and plant performance. Carbon and nitrogen cycling capacities were evidenced by genes involved in organic matter decomposition and nitrogen transformations by bacteria and archaea, including nif genes for biological N_2_ fixation, *amoA* for nitrification, and *nirK/nirS*, *norB*, and *nosZ* for denitrification, collectively indicating an active microbial contribution to nitrogen turnover and bioavailable nitrogen pools in the soil [65]. P mobilization potential was supported by the presence of genes such as *phoA/phoD* and other phosphatases, as well as phosphate transport and regulatory genes including *pstSCAB*, *phoB*, *phoR*, and *phoU*, which are implicated in phosphate scavenging and solubilization under nutrient‐limited conditions [66]. Phytohormone production capacity was suggested by tryptophan‐dependent indole‐3‐acetic acid biosynthesis genes, including *ipdC*, *iaaM*, and *iaaH*, which are known to influence root elongation and modulate plant–microbe signaling [67, 68]. Stress‐alleviation mechanisms were indicated by genes associated with osmoprotection and general stress tolerance, including those involved in trehalose and glycine betaine biosynthesis, as well as *acdS* encoding ACC deaminase, which can attenuate stress‐induced ethylene levels and thereby promote plant growth under adverse environmental conditions [69]. Taken together, these functional traits indicate a rhizosphere microbiome with the potential to enhance nutrient acquisition, regulate root development, and increase plant stress resilience.

## 3. Metagenomic Approaches for Revealing Microbiome Functions and Diversity

The field of microbial community research has experienced a transformative shift as a result of progress in NGS techniques. These innovations have empowered researchers to investigate the structure, diversity, and functional aspects of microbiomes with unprecedented precision. Two primary methodologies, amplicon sequencing (which uses markers such as 16S rRNA for studying bacteria as well as archaea and internal transcribed spacer (ITS) for fungal characterization) and shotgun metagenomics (Figures 1 and 2), constitute the foundational techniques utilized in the majority of microbiome investigations [70]. Complementing these methods is a swiftly advancing network of computational tools, functional profiling methods, and integrative multiomics strategies, which collectively extend the boundaries of microbiome science (Figure 3).

**Figure 1:**
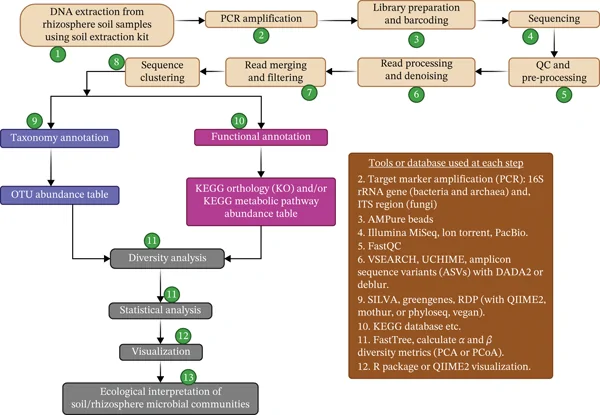
Overview of amplicon sequencing workflow for rhizosphere and soil microbiome profiling: from soil sampling and DNA extraction to microbial community analysis and ecological interpretation. Created by the authors with BioRender (https://www.biorender.com). Note: The amplicon sequencing workflow for soil and rhizosphere microbiome profiling involves the extraction of microbial DNA from bulk soil or rhizosphere samples, followed by PCR amplification of targeted taxonomic marker regions, including the 16S rRNA gene for bacteria and archaea and the internal transcribed spacer (ITS) region for fungi. The amplified marker genes are then sequenced and processed through bioinformatic pipelines for quality filtering, taxonomic assignment, microbial community characterization, and ecological interpretation. Taxonomic classification relies on marker‐gene reference databases such as SILVA for bacterial and archaeal communities and UNITE for fungal communities. Functional predictions derived from amplicon datasets may use metabolic reference databases such as KEGG through tools including PICRUSt2 and Tax4Fun2.

**Figure 2:**
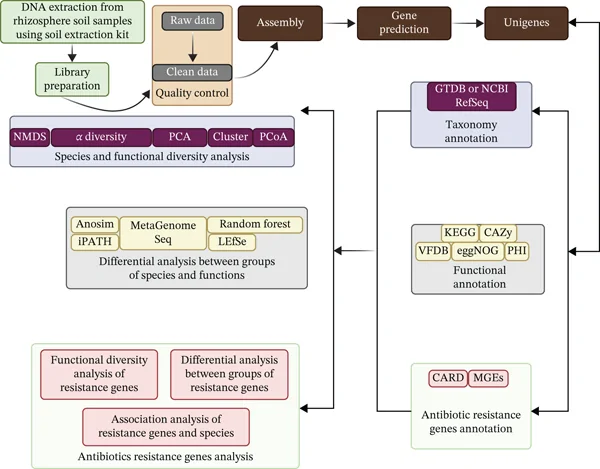
Shotgun metagenomic workflow for rhizosphere and soil microbiome characterization: from microbial DNA extraction to taxonomic profiling, functional annotation, and ecological interpretation. Created by the authors with BioRender (https://www.biorender.com). Note: Shotgun metagenomic workflows for soil and rhizosphere samples are broadly standardized, but interpretation depends on the DNA source, microbial composition, and reference databases. Taxonomic profiling uses genome databases such as GTDB and NCBI RefSeq, whereas functional annotation relies on databases such as KEGG, EggNOG (evolutionary genealogy of genes: Nonsupervised Orthologous Groups), and CAZy with tools like HUMAnN3 and DIAMOND.

**Figure 3:**
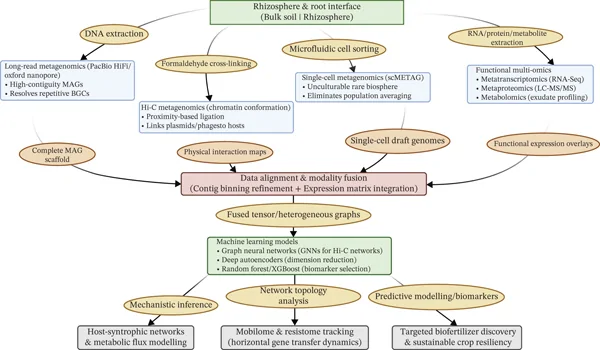
An integrated pipeline combining emerging single‐cell, spatial (Hi‐C) metagenomics, and functional multiomics with machine learning at the rhizosphere‐root interface. Created by the authors with BioRender (https://www.biorender.com).

### 3.1. Shotgun Sequencing Techniques and Amplicon Sequencing (16S/ITS): Comparison of Their Strengths and Limitations

Amplicon sequencing (Figure 1), which focuses on the 16S rRNA gene (which serves as a critical molecular marker for the characterization and identification of bacterial and archaeal communities) and the ITS region, which serves as a critical molecular marker for fungal communities, has been a fundamental technique in microbial soil ecology [71]. This approach involves amplification via polymerase chain reaction (PCR) of conserved genetic markers, followed by sequencing and subsequent taxonomic classification. Its advantages include its cost‐effectiveness, scalability, and capacity to generate reliable genus‐level and occasionally species‐level taxonomic profiles across extensive sample collections, especially in resource‐constrained environments [72].

Amplicon sequencing, despite its effectiveness, has significant limitations. The limitation of this method is associated primarily with the selection of primers and the subsequent amplification process in polymerase chain reaction (PCR), which may alter the representation of particular taxa [73]. Its taxonomic resolution capability is predominantly constrained to the genus level, rendering species‐level identification frequently unreliable, particularly for phylogenetically proximate organisms [72]. Furthermore, amplicon sequencing provides only indirect inferences about functional potential, as it does not capture the full complement of gene representation within the community [74].

In contrast to targeted approaches involving amplicon sequencing, shotgun metagenomics (Figure 2) involves sequencing all DNA present within a given sample, thereby encompassing not only bacterial and archaeal DNA but also that of viruses, fungi, and other eukaryotic organisms [13]. This methodology facilitates extensive taxonomic profiling with significantly higher resolution, often extending to the species or even strain level, and facilitates the direct evaluation of functional genes and metabolic pathways. Shotgun metagenomics is particularly efficient in identifying rare or low‐abundance taxa and in characterizing the functional diversities of microbial communities [75]. Nonetheless, the limitations associated with shotgun metagenomics include its increased financial cost, increased computational requirements, and the need for deeper sequencing efforts to obtain adequate coverage, particularly in complex or low‐biomass samples [76]. The data analysis process is more intricate, necessitating robust bioinformatics pipelines and considerable computational resources [77].

Comparative analyses have demonstrated that although both methods can yield similar genus‐level profiles for bacterial, archaeal and fungal communities, shotgun metagenomics consistently outperforms amplicon sequencing in achieving higher species‐level resolution, identifying rare taxa, and predicting functional genes [78]. Compared with 16S/ITS amplicon sequencing, shotgun metagenomics generally provides higher species‐ and strain‐level resolution, improved detection of rare and nontarget taxa (including viruses), and direct, more accurate prediction of functional genes and pathways [13]. Nevertheless, for fungal studies, the concordance between shotgun metagenomics and ITS amplicon sequencing is frequently limited, underscoring the necessity for methodological selection tailored to specific research objectives and target organisms [79].

However, shotgun metagenomics does not consistently outperform amplicon sequencing, as performance varies with sample type, read depth, and taxonomic level. Tessler et al. [80] reported that amplicon sequencing recovered more than 27% more families and more phyla than shotgun sequencing did in Brazilian river samples, contradicting the superiority of shotgun sequencing. Usyk et al. [79] reported equivalent bacterial genus‐level accuracy for both methods, and Park et al. [81] noted that the advantage of the functional resolution of a shotgun method is “strongly constrained by microbial read depth” in host‐rich samples, so low microbial read depth limits functional insights when host DNA is abundant.

Figure 1 above illustrates the workflow of amplicon sequencing, which constitutes a robust and standardized framework for characterizing microbial communities, systematically integrating wet‐laboratory sample preparation with advanced downstream bioinformatic analysis [82]. Beginning with DNA extraction and marker–gene PCR amplification followed by high‐throughput sequencing, the pipeline facilitates highly targeted data acquisition. The bioinformatic core incorporates stringent quality control, deionizing, and sequence clustering procedures (employing tools such as FastQC, DADA2, and VSEARCH) to minimize PCR‐derived artifacts and sequencing errors, thereby enabling the generation of reliable amplicon sequence variants (ASVs) or operational taxonomic units (OTUs). In parallel, taxonomic assignment against curated reference databases (e.g., SILVA, Greengenes) and functional inference via Kyoto Encyclopedia of Genes and Genomes (KEGG)‐based annotation support a comprehensive, dual‐perspective analysis that resolves both community composition (“who is present”) and putative metabolic potential (“what functional capacities are encoded”). The subsequent integration of these complementary abundance matrices into ecological diversity indices and statistical visualization workflows provides a rigorous and reproducible framework for extracting biologically meaningful insights from complex metagenomic datasets.

Figure 2 schematically depicts a shotgun metagenomics workflow that employs an assembly‐first strategy to characterize the taxonomic composition, functional repertoire, and antibiotic resistance determinants of microbial communities [83]. Following initial quality control to obtain high‐fidelity sequencing reads, de novo assembly and subsequent gene prediction are performed to generate a nonredundant catalog of unigenes. This centralized gene catalog is subsequently analyzed via three parallel annotation pipelines: one for taxonomic assignment via the Micro NR database; another for metabolic and phenotypic profiling on the basis of core functional resources (using KEGG, CAZy, eggNOG, VFDB, and PHI); and a third for resistome analysis focused on antibiotic resistance genes and mobile genetic elements (CARDs and MGEs).

Downstream statistical analyses integrate these multilayered annotations to elucidate community ecological dynamics. In particular, species‐ and function‐level matrices are subjected to multivariate ordination and diversity analyses (NMDS, PCoA, PCA, and alpha diversity metrics) in conjunction with nonparametric and machine learning‐based differential abundance testing (LEfSe, random forest [RF], MetaGenomeSeq, Anosim, iPATH) to identify discriminant biomarkers among experimental groups. In parallel, the resistome‐specific branch yields a focused characterization of antibiotic resistance, delineating the diversity, differential abundance, and putative host–species associations of resistance determinants, thereby providing a comprehensive assessment of community functional potential and biosafety risks.

#### 3.1.1. Factors Influencing the Choice Between Amplicon and Shotgun Sequencing in Soil and Rhizosphere Metagenomic Studies

Soil and rhizosphere samples present specific methodological challenges for metagenomic analyses that directly influence sequencing strategies. Amplicon sequencing of taxonomic marker genes (e.g., 16S rRNA, ITS) is often preferred for soil community surveys because it is sensitive for detecting microbial taxa in complex microbial assemblages, is cost‐effective for large sample sets, and benefits from comprehensive 16S reference databases covering diverse soil microbial communities [84].

In contrast, shotgun metagenomic sequencing not only detects microbial taxa but also offers higher functional resolution for soil microbiomes (e.g., nutrient cycling genes and plant growth‐promoting traits). However, it requires high‐quality microbial DNA and is hindered by high organic matter and humic substances, which interfere with DNA extraction and sequencing [85].

However, choosing between amplicon‐based methods and shotgun metagenomic sequencing for soil microbiome studies depends on soil traits and study design. Amplicon sequencing (e.g., 16S rRNA, and ITS) is preferred when the focus is on taxonomic profiling of highly diverse soil communities with hundreds of taxa per gram; when many samples must be processed on a limited budget (e.g., greenhouse trials with many replicates); when detecting low‐abundance taxa is important, targeted PCR improves sensitivity; and when comprehensive, well‐curated marker gene databases (e.g., SILVA and Greengenes) with good coverage of soil bacteria are available [86].

However, amplicon sequencing has important limitations for soil microbial communities, including PCR bias from primer mismatches with uncharacterized taxa and an inability to directly infer functional potential (e.g., genes for nutrient cycling or plant growth promotion) [87]. Thus, shotgun metagenomic sequencing is preferred when (1) functional annotation is the main goal, such as detecting and characterizing genes for phosphate solubilization, indole‐3‐acetic acid (IAA) biosynthesis, or nitrogen fixation in biofertilizer development; (2) strain‐level resolution is needed to distinguish closely related rhizosphere isolates; and (3) high‐quality metagenome‐assembled genomes (MAGs) are needed for genome‐resolved and comparative analyses [88].

The main drawbacks of shotgun metagenomics include high organic matter and humic substances that inhibit library preparation and sequencing and abundant host DNA in rhizosphere samples, which reduces microbial sequencing depth and often requires host DNA depletion or microbial DNA enrichment [89]. In rhizosphere microbiome studies of plant–microbe interactions and biofertilizer effects, amplicon sequencing is generally best for broad, cost‐effective taxonomic profiling, whereas shotgun metagenomics is preferable when resolving functional mechanisms of plant growth promotion at the gene, pathway, and metabolic levels.

### 3.2. Computational Tools and Pipelines for Metagenomics Analysis

The explosion of microbiome data has spurred the advancement of a wide spectrum of computational tools and analytical pipelines, each specifically designed to address distinct sequencing techniques and research investigations.

Amplicon sequencing uses a suite of computational tools (pipelines) that are extensively applied within the tools, such as QIIME2, which serves as a modular and extensible platform facilitating quality control, taxonomic assignment, diversity analysis, and visualization (Figure 1). QIIME2 accommodates both ASV workflows and OTU workflows, and it provides plugins for denoising, including DADA2 and Deblur, as well as integration with multiple databases of reference sequences such as SILVA and Greengenes [90]. Similarly, Mothur is a well‐established pipeline specifically designed for the processing of 16S rRNA gene sequences, offering capabilities for robust clustering, chimera detection, and taxonomic profiling [91]. DADA2, a high‐resolution denoising algorithm, enhances the sensitivity and specificity of amplicon data by inferring exact sequence variants [92].

However, in shotgun metagenomics sequencing, several pivotal computations (pipelines) are utilized for the analysis of metagenomic data (Figure 2). Among these, MG‐RAST serves as an automated platform for facilitating both taxonomic and functional annotations through the employment of diverse reference databases [93]. Likewise, MEGAN offers a robust framework for the interactive exploration and scrutiny of extensive metagenomic datasets, enabling both taxonomic and functional classification while seamlessly integrating with high‐throughput aligners such as DIAMOND [94]. In the same vein, Kraken2 is recognized for its exceptional speed as a k‐mer‐based classifier, delivering high‐accuracy taxonomic assignments for both amplicon and shotgun sequence data [95]. Moreover, HUMAnN3 is proficient at functional profiling, reconstructing metabolic pathways from metagenomic or metatranscriptomic data by mapping sequence reads to gene families and pathways through databases such as KEGG, UniRef, and MetaCyc [96, 97]. Similarly, PICRUSt2 is tailored for predictive functional profiling via 16S rRNA gene data, inferring the potential functionality of microbial communities by correlating taxa with known gene content from reference genomes [74].

However, the choice of computational pipelines and reference databases is a critical factor that can substantially affect the outcomes of microbiome research, with the SILVA and RefSeq/Kraken2 standard libraries documented to offer increased accuracy for taxonomic assignments relative to the Greengenes database. Furthermore, tools initially developed for whole‐genome metagenomics analyses, exemplified by tools such as PathoScope and Kraken2, have demonstrated superior performance with 16S amplicon data, occasionally surpassing traditional amplicon‐centric methodologies [72].

### 3.3. Predictive Functional Profiling: Transitioning From Taxonomic Classification to Functional Assessment

The primary objective of microbiome research is to move beyond basic taxonomic composition and elucidate the functional capabilities of microbial communities (Table 1). Predictive functional profiling efforts seek to bridge this gap by leveraging taxonomic data to decipher the incidence of genes and metabolic pathways.

Tools such as PICRUSt2, Tax4Fun2, and FAPROTAX in amplicon data facilitate the prediction of functional profiles through the mapping of observed taxa to reference genomes and their annotated gene content within databases such as eggNOG, COG (Clusters of Orthologous Groups), and KEGG (Figure 1) [98]. These predictions are instrumental in formulating hypotheses concerning microbial community function, particularly when shotgun metagenomics is not feasible. However, the precision of these predictions is constrained by the quality of taxonomic assignments, the completeness of reference databases, and the presumption that phylogenetically similar taxa possess analogous functional repertoires. In agriculture, 16S rRNA gene sequencing elucidates the compositional structure of microbial diversity but is not sensitive enough to identify crucial functional potential or ecologically relevant microbiome impacts that affect soil fertility and crop growth, necessitating careful interpretation [99].

Conversely, shotgun metagenomics, a technique that involves sequencing the whole complement of genes directly within a community, affords a more dependable and exhaustive perspective of functional potential (Figure 2) [75]. Sequencing reads can be aligned to gene databases and metabolic pathways via the COG, eggNOG, and KEGG databases, facilitating the detailed characterization of the community of microorganisms and their metabolism and functional diversity [100]. This approach is efficient when integrated with pathway‐centric tools such as HUMAnN3, which enables profiling of the abundance and completeness of metabolic pathways across samples [1, 101].

### 3.4. Multiomics Data Integration for Machine Learning‐Powered (AI) Computational Modeling

In the rapidly advancing field of microbiome research, there has been a notable shift toward integrative, multiomics methodologies that encompass not only the genetic potential of communities of microorganisms but also their dynamic functions and interactions with both the host and the environment (Figure 3) [102, 103]. Metatranscriptomics plays a pivotal role by sequencing the total RNA from a community, thus elucidating gene expression and active metabolic processes. This approach offers an essential understanding of which genes are actively transcribed under specific environmental conditions, thereby offering a dynamic perspective on community function that complements the static gene inventories yielded by metagenomics [104]. By further extending this analysis to the protein level, metaproteomics facilitates characterization of the expressed proteome within a microbial community by identifying and quantifying microbial proteins [105]. Metaproteomics offers direct evidence of functional activity and can reveal posttranslational modifications and protein–protein interactions. Several sophisticated software tools, such as ProPHAnE, eggNOG‐mapper, MetaProteomeAnalyzer, MetaGOmics, MEGAN, and Unipept, facilitate functional profiling and comparative analysis of metaproteomic data, each exhibiting unique strengths in terms of sensitivity, specificity, and functional group coverage [106]. Concurrently, metabolomics analyzes the metabolites (intermediate products) synthesized or produced by microbiota, thereby offering a holistic perspective of the biochemical products and ecological impacts of microbiomes in the soil [107]. However, combining metaproteomics metagenomics, metatranscriptomics, and metabolomics, referred to as “multiomics”, with soil sciences enables a holistic understanding of the structure, function, and dynamics of the rhizosphere microbiome [102, 103].

However, multiomics data integration into soil sciences poses significant analytical obstacles, including data normalization, dimensionality reduction, and the necessity for robust statistical and computational frameworks (Figure 3) [108]. Within this framework, computational learning algorithms (MLs) and artificial intelligence (AI) increasingly assume central roles. ML algorithms can handle extensive, high‐dimensional datasets to discern complex patterns, identify biomarkers, and predict functional or disease outcomes (Table 2) [128]. In particular, deep learning models have demonstrated efficiency in automating feature extraction, modeling nonlinear relationships, and integrating heterogeneous data types for agricultural prediction, as evidenced in their application in wheat yield prediction studies by Ashfaq et al. [129].

**Table 2:** Overview of the predictive model for agricultural management.

Model type	Target variable	Best *R* ^2^/accuracy	Key microbial features	Citations
i. XGBoost	Soil health index (MISH)	Strong correlation	Functional gene profiles	[109]
ii. Two‐step ML (random forest + clustering)	Crop health (NDVI)	Exploratory promising	Fungal soil microbiome taxa	[110]
iii. Locally trained ML	Maize yield intrafield variation	High prediction accuracy	Local microbial yield‐associating signatures	[111]
iv. 16S rRNA sequencing + ML	Soil biological health	High potential utility	Soil bacterial taxa	[112]
v. Random forest (RF), SVM	Soil health metrics (biological, chem., and physical)	0.8	Pyrinomonadaceae, Nitrososphaeraceae, *Candidatus Udaeobacter*	[112]
vi. RF, XGBoost, BPNN	Crop yield	0.813–0.818	Bacterial biomarkers, soil organic carbon	[113]
vii. Deep learning (DL)	Fungal disease occurrence	> 90% accuracy	Chitinophagaceae, *Nocardioides*, *Sphingomonas*	[114]
viii. Random forest	Soil quality, land use, and soil variables	Up to 0.79	Top 15 OTUs, pH, nutrient concentrations	[115]
ix. Hybrid (Microbial‐explicit + RF)	Soil organic carbon (SOC)	0.84	Microbial CUE, biomass carbon, net primary productivity	[116]
x. Random forest	Crop productivity	0.79	*Actinomycetiales*, *Nostocales, Rhizobiales*	[117]
xi. Regression	Wheat yield, grain quality	0.64–0.90	Fungal diversity, nitrification genes	[118]
xii. RF, Bayesian NN	Potato yield, disease	High F1 scores	Paenibacillaceae, Moraxellaceae, Syntrophaceae	[119]
xiii. Random forest	Fusarium wilt disease occurrence	> 80% accuracy	*Streptomyces*, Bradyrhizobiaceae, Comamonadaceae	[120]
xiv. Regression	Crop yield, ecosystem multifunctionality	High predictive power	Microbial diversity at broad phylogenetic scales	[121]
xv. Machine learning	Disease‐suppressive soils	High accuracy	54 biomarker genera (e.g., *Kribbella*, *Nocardioides*)	[34]
xvi. Cubist model	Soil microbial communities	> 90% effectiveness	PLFA, ammonium‐N, phosphorus, pH	[122]
xvii. RF, stepwise, exhaustive	Maize growth response to AMF inoculation	86% variation explained	Pathogenic fungi abundance, cMIC, mineralized nitrogen	[123]
xviii. RF, neural Network	Methane yield (anaerobic digestion)	0.82	*Chloroflexi*, *Actinobacteria*, *Proteobacteria*	[124]
xix. Tax4Fun, PanFP	Functional gene prediction	Qualitative match	Enzyme‐coding genes, 16S rRNA‐based predictions	[7, 8]
xx. Machine learning (CheckM2)	Genome quality (MAGs)	High accuracy	Multicopy genes, pathway modules	[125]
xxi. Linear Regression	Microbial indicators vs. crop yield	Reference values	MBC, basal respiration, enzyme activities	[126]

However, AI‐driven approaches have been employed to forecast host plant phenotypes and assess disease risk in soils by analyzing microbiome profiles (Table 2). These approaches facilitate the identification of microbial biomarkers pertinent to disease diagnosis and the detection of microbial resistance genes [130]. Additionally, AI‐driven models are utilized to stimulate microbial interactions and community dynamics. Furthermore, these methods enable the integration of multiomics data, allowing for exhaustive functional profiling [131]. The development of specialized ML software and frameworks facilitates careful preprocessing, feature selection, model validation, and transparency, with tools such as RFs, logistic regression, and individual conditional expectation (ICE) plots providing both predictive power and biological insight in the soil sciences [132].

#### 3.4.1. Emerging Metagenomics Approaches

Recent progress in metagenomic sequencing has produced multiple new methods that improve the characterization of microbial communities compared with conventional techniques.

Long‐read metagenomic sequencing employing Pacific Biosciences (PacBio) HiFi and Oxford Nanopore technologies (ONT) produces sequence reads on the scale of several kilobases (Figure 3). This increased read length facilitates the reconstruction of more contiguous and complete genome assemblies, enhances the resolution of repetitive and structurally complex genomic regions, and improves taxonomic discrimination down to the strain level, particularly in comparison with conventional short‐read Illumina sequencing approaches [133, 134].

Third‐generation long‐read metagenomic sequencing overcomes the assembly limits of short‐read platforms in heterogeneous samples and is transforming agricultural microbiome research. PacBio HiFi and ONT generate long (> 10 kb), continuous reads that avoid the fragmentation inherent to short‐read workflows. PacBio HiFi uses circular consensus reads to reach > 99.9% accuracy, matching short‐read fidelity while preserving structural continuity. This enables tools such as metaMDBG and hifiasm‐meta to reconstruct complete, circular, single‐contig MAGs from uncultured microbes [135, 136]. Long reads also resolve repetitive regions, directly linking specialized functional gene clusters, such as plant‐growth‐promoting (PGP) operons and antibiotic resistance genes, to their host organisms. Combined with advanced DNA extraction methods, these long‐read approaches provide unprecedented resolution of the multikingdom networks that drive nutrient cycling at the crop–root interface [137] (Figure 3).

Single‐cell metagenomics (scMETAG), another emerging technique, involves the physical isolation and genomic sequencing of individual microbial cells within complex communities, thereby circumventing the requirement for bulk community DNA assembly (Figure 3). This approach facilitates the reconstruction of high‐resolution genomes from rare or low‐abundance taxa that are frequently underrepresented or masked in conventional, population‐averaged metagenomic datasets [138]. scMETAG, enabled by long‐read sequencing, overcomes the loss of cellular context in bulk community extractions [139].

By physically separating individual microbial cells from complex samples via microfluidic or droplet‐based platforms, single‐cell metagenomic methods characterize genomes at single‐cell rather than community‐bin resolution (Figure 3). This is crucial in complex microbiomes, where single‐cell sequencing is difficult to apply uniformly because cells contain little DNA, and taxa differ in their cell wall structure and lysis requirements [140]. In the rhizosphere and soil, single‐cell genomics broadens microbial genome catalogs and, when combined with metagenomic data, reveals strain‐level patterns and dynamics in the root zone. It is especially effective for obtaining diverse, high‐quality genomes from soil and rhizoplane communities, where standard approaches still struggle. However, single‐cell technologies remain limited by low encapsulation efficiency, amplification bias, and poor recovery of some biofilm‐associated microbes [141].

Similarly, high‐throughput chromosome conformation capture metagenomics (Hi‐C) combines shotgun sequencing with proximity ligation to link DNA fragments that are adjacent inside intact cells, adding spatial context to metagenomic data (Figure 3). This approach improves contig binning and genome‐resolved analyses because Hi‐C contacts connect fragments from the same cell and generate contig contact matrices that guide binning [142, 143]. In wheat rhizosphere studies, short‐read–only binning often results in highly contaminated bins, whereas the addition of Hi‐C data improves MAG quality and reduces the number of chimeras [142]. Pairing long‐read sequencing with Hi‐C further enhances MAG recovery, especially when multiple binning methods are combined rather than used in isolation [136, 143]. Hi‐C also helps link mobile genetic elements to their hosts, as proximity‐based contacts associate plasmids, phages, and ARG‐carrying elements with likely hosts [144]. For rhizosphere‐focused applications, Hi‐C data can greatly improve genome binning and host‐linkage inference, but the resulting assemblies and associations are most reliable when systematically benchmarked, carefully filtered, and integrated with complementary sequencing and analytical methods (Figure 3) [141].

Together, these methodologies overcome key limitations of conventional metagenomics, highly fragmented assemblies, poor resolution of closely related strains, and bias toward abundant taxa, enabling more comprehensive characterization of plant–microbe interactions and more accurate assessment of microbial functional potential in agricultural ecosystems.

## 4. Applications of Metagenomic Tools in Sustainable Agriculture

Metagenomic tools have emerged as pivotal and cutting‐edge tools in the advancement of agricultural sustainability, providing an unparalleled understanding of the diversity, functionality, structure, and makeup of soil and plant‐associated microbial communities. These tools have facilitated the direct sequencing and analysis of genetic materials obtained from environmental samples, thereby circumventing the limitations inherent in traditional culture‐based techniques. This approach reveals the extensive array of microorganisms that are integral to soil health, plant growth, and ecosystem resilience [13].

Metagenomics presents a robust scientific method for achieving SDG 2 by 2030 by facilitating innovative solutions for food security and sustainable agriculture. This involves developing crops that are resilient to climate change, increasing drought and salinity tolerance, and directly supporting SDG2′s goals of eradicating hunger and promoting sustainable agriculture to ensure food availability for everyone [145].

The utilization of metagenomics tools, including QIIME2, MG‐RAST, and Kraken2, in sustainable agriculture has significantly enhanced the monitoring of soil health. As a dynamic ecosystem, soil harbors an intricate community of beneficial microorganisms that facilitate nutrient cycling, decomposition of organic matter, and contaminant degradation (Table 1) [11]. Metagenomics provides a comprehensive framework for characterizing these microbial communities by identifying both the taxonomic constituents and functional genes within the soil matrix, particularly the rhizosphere of the soil (Table 1) [146]. Similarly, through the monitoring of microbial indicator processes related to soil fertility, such as those involved in fixing AN, solubilizing phosphate, and decomposing organic matter, researchers and farmers can evaluate the biological quality of soils with unprecedented precision [147]. This approach has also revolutionized the detection of alterations in the community of microorganisms resulting from land management practices, environmental pollution, or climatic changes, thereby offering early indicators of soil degradation or contamination. Robust microbial indicators through metagenomic approaches are now critical for sustainable land management and conservation strategies, as they ensure the sustainable maintenance of soil quality and productivity [148].

Similarly, metagenomics tools in agriculture have fundamentally transformed the formulation of biofertilizers, which are microbial preparations used to augment and ameliorate crop development and soil nutrient status. Through the analysis of genetic material from soil and rhizosphere ecosystems, metagenomics facilitates the identification of beneficial microorganisms with traits related to nutrient mineralization and fixation and the production of phytohormones favorable for plant growth, thereby decreasing the dependence on chemical fertilizers and mitigating environmental consequences [13, 149]. Additionally, metagenomics supports the discovery of genes associated with these beneficial traits (Table 1), advancing bioengineering and synthetic biology strategies to engineer next‐generation biofertilizers with improved efficacy and customized functionalities [32]. The integration of metagenomics with high‐throughput screening and other omics technologies is promoting the advancement of sustainable, environmentally favorable solutions to address the growing global food demand [146].

Furthermore, metagenomic tools such as CARD (antibiotic resistance gene database), MetaGeneMark and HUMAnN offer robust potential in biological control and disease suppression for the detection of antagonistic microorganisms and functional genes involved in pathogen suppression [150, 151]. Soil‐borne pathogens and pests present major risks to crop production. However, metagenomics enables the characterization of microbial gene clusters and taxa linked to natural disease resistance, such as those encoding antibiotics, lytic enzymes, and siderophores [147]. Elucidating the mechanisms by which beneficial microorganisms inhibit pathogen proliferation enables researchers to devise effective biocontrol agents and disease‐suppressive soil amendments, thereby reducing the dependence on synthetic agrochemicals and promoting agroecosystem sustainability [28]. Furthermore, functional metagenomics, coupled with network analysis, bolsters the targeted recruitment or augmentation of beneficial microbes within the rhizosphere, thereby enhancing plant protection and contributing to overall ecosystem health (Table 1) [152].

However, the application of metagenomic tools in climate‐smart agriculture is gaining significant attention, particularly because of the pivotal roles played by soil microbes in processes such as carbon sequestration, mitigation of greenhouse gas emissions, and bolstering crop tolerance against stressors such as salinity and drought. By profiling soil microbial communities, researchers can monitor microbes involved in the regulation of greenhouse gases, including methanotrophs and denitrifiers, which are critically important in reducing methane and emissions of nitrous oxide from agricultural soils [153].

Similarly, the identification of metabolic pathways that play a role in stabilizing soil organic carbon, reducing greenhouse gas emissions, and improving the efficiency of nutrient and water usage has been facilitated by the metagenomic approach (Table 1). These insights are instrumental in shaping management practices and interventions, including the introduction of specific microbial consortia or soil amendments aimed at fostering climate resilience and sustainability [154]. Through metagenomics, researchers can devise ecosystem restoration strategies that support healthy soils, robust crop production, and environmental conservation amidst climate change challenges [145].

A critical implementation of metagenomics in sustainable agriculture is the identification of beneficial microbes and their functional genes. Through the use of advanced sequencing and bioinformatics, scientists can identify novel microorganisms and enzymes involved in plant growth‐promoting activities, all of which contribute to increased crop yields and diminished dependency on chemical fertilizers [152]. Metagenomic pipelines such as PGPg_finder enable the detection of functional genes involved in promoting plant growth, facilitating the development of tailored biofertilizers and biopesticides that are both environmentally sustainable and tailored to specific crops or soil conditions [155].

Additionally, metagenomic studies have also been helpful in bioremediation by characterizing microbes capable of degrading chemicals used in pest control, toxic metals, and various contaminants, thus contributing to the restoration of contaminated soils [156]. The incorporation of metagenomic data with other omics technologies and AI augments the detection of metabolic pathways and novel genes, thereby paving the way for innovative solutions to the pressing issues of food security, environmental pollution and conservation, and climate resilience [157].

## 5. Metagenomic Insights and Applications in Diverse Cropping Systems for Agricultural Sustainability

Metagenomic analyses have been successfully employed across diverse cropping systems, including maize, leek, wheat, soybean, oil palm, and walnut orchards (Table 1). Adedayo et al. [158] demonstrated the efficiency of shotgun metagenomics in characterizing the rhizosphere microbiomes of healthy and diseased tomato plants. The investigation revealed that the rhizosphere of healthy tomato plants presented increased microbial diversity and abundance relative to diseased tomato plants, with bacterial taxa, including Proteobacteria, Myxococcota, and Actinobacteria, being integral to crop health and resilience. The use of metagenomics has been successful in the profiling of beneficial microbes and their functional genes and functions in maize plants [39, 40], rice [159], and leek [9].

Comparative analyses between soils treated with chemical fertilizers and those treated with biofertilizers have yielded significant insights. Shotgun metagenomics analysis of maize roots cultivated under long‐term fertilization regimes revealed that although the overall abundance of microbial functional genes was comparable, the diversity and specific metabolic pathways exhibited marked variation across different treatments [39, 40]. Notably, organic fertilization was associated with an increased abundance of beneficial functional diversity related to nutrient cycling and plant growth promotion, indicating that biofertilizers enhance soil health and sustainability more effectively than chemical fertilizers do. Additional studies have shown that the excessive use of agrochemicals (synthetic fertilizers) can disrupt the structure of microbial communities, decrease beneficial interactions, and lead to increased soil acidification. In contrast, biofertilizers and organic amendments support a more balanced and resilient soil microbiome, as illustrated by [13, 16] on leek (*Allium ampeloprasum*). These conclusions are based on metagenomic analyses of rhizosphere soils that compared taxonomic composition, functional gene repertoires, and predicted metabolic pathways between chemical fertilized, biofertilizer treated soil, and bulk soils as control treatments.

Metagenomic data, in conjunction with bioinformatics tools, have facilitated the characterization of microbial genes associated with plant growth promotion and stress tolerance, which serve as robust predictors of plant productivity and soil quality. Precisely, raw metagenomic reads from rhizosphere soil are subjected to quality‐control filtering and assembled into contigs using tools such as MEGAHIT, followed by gene prediction with Prodigal. The predicted genes are annotated against functional databases, including KEGG, EggNOG, and CAZy, using alignment tools such as DIAMOND [160]. These analyses enable the identification of microbial functional potential associated with rhizosphere processes, including nutrient cycling, plant growth promotion, stress adaptation, and microbial interactions.

Through comprehensive metagenomic analyses, several key microbial communities have been identified, notably PGPR, including the genera *Bacillus, Pseudomonas, Burkholderia, Azospirillum,* and *Trichoderma*, as well as nitrogen‐fixing bacteria, including *Azotobacter* and *Rhizobium*, alongside P‐solubilizing microbes. These microbiomes have been shown to increase nutrient acquisition, synthesize phytohormones, and suppress phytopathogenic organisms, thereby facilitating plant health and increasing agricultural productivity [161].

Endophytic bacteria and fungi, often unculturable by traditional methods, have been characterized via metagenomics. These include members of Actinobacteria, Proteobacteria, and Firmicutes, which contribute to tolerance to stress and resistance to diseases in plants [162].

However, across diverse cropping systems, metagenomic studies have consistently shown that bacteria constitute the majority of soil microbiomes. Bacterial phyla dominate rhizosphere and bulk soils in crops such as carrots and leeks, a trend also observed in broader agroecosystem surveys [13, 163]. This dominance likely stems from the crucial ecological roles of bacteria in nutrient cycling, decomposition, and plant‐associated functions within agricultural soils. Furthermore, bacterial communities are highly responsive to management practices, including crop rotation, residue return, fertilization, and the choice between organic and conventional farming [16, 63, 64].

In contrast, the significantly lower detection of fungi and archaea in the metagenomic datasets does not necessarily imply their unimportance. Instead, many studies report only a limited number of fungal and archaeal phyla or very low relative abundances. This discrepancy can be attributed to methodological biases, including those arising from DNA extraction, primer selection, PCR amplification, sequencing platforms, bioinformatic pipelines, and incomplete database resolution, all of which can systematically underrepresent certain taxa compared with bacteria [164].

Shotgun sequencing has identified several agriculturally important fungal and archaeal taxa linked to phosphate solubilization, nitrification, and broader nitrogen turnover in soils [165]. The strongest evidence points to archaeal Thaumarchaeota/Nitrososphaeraceae in ammonia oxidation and to fungal groups such as Rhizophagus, Funneliformis, *Trichoderma*, Penicillium, and Aspergillus in rhizosphere functioning, P uptake partnerships, and plant‐beneficial interactions [15, 166] (Table 1).

Although shotgun metagenomics remains the most informative approach for linking soil microbial diversity to function across cropping systems, the evidence suggests that interpretations of low fungal and archaeal recovery should be made cautiously. Ideally, these analyses should be complemented by targeted markers, such as ITS for fungi and 16S‐based archaeal analyses, to more accurately capture the full spectrum of the soil microbiome.

## 6. Integrating Predictive Models With Microbial Indicators for Sustainable Agriculture

Progress in predictive modeling has further enhanced the association between microbial functions and crop yield and soil health. Current developments in metagenomics and machine learning have enabled the conception of predictive models that establish correlations between soil microbial functions in relation to crop yield and soil health, thereby presenting transformative potential for sustainable agricultural practices (Table 2).

These models utilize high‐throughput sequencing data alongside advanced algorithms to elucidate the intricate relationships among microbial communities, soil characteristics, and agricultural productivity [167]. A significant breakthrough has been the deployment of machine learning models such as RF, extreme gradient boosting (XGBoost), and support vector machines (SVMs) to forecast crop yield and soil health metrics on the basis of microbiome data (Table 2). Empirical evidence has demonstrated that the RF and XGBoost models, when calibrated with bacterial community profiles and soil physicochemical information, can predict crop yields with notable accuracy, with *R* ^2^ values ranging from approximately 0.81–0.82 [168]. The *R* ^2^ coefficient functions as an essential statistical metric that quantifies the proportion of variance elucidated by predictive models within the context of agricultural sustainability research. This provides an indication of the degree to which regression models effectively encapsulate agricultural phenomena [114].

Bacterial biomarkers have emerged as crucial predictors, contributing to as much as 40% of model importance. The exclusion of these biomarkers from the models has resulted in a substantial decrease in predictive efficacy, highlighting their indispensable role in yield prediction. Similarly, soil health metrics, which encompass biological, chemical, and physical attributes, can be inferred from microbiome data, with *R* ^2^ values reaching 0.8. Biological health assessments, particularly those pertaining to microbial activity and diversity, are exceptionally well predicted, and these models have demonstrated generalizability across independent datasets [113].

Notably, the characterization of microbial functional gene profiles, such as those involved in the cycling of nutrients, has led to the emergence of robust indices, including the Molecular Index of Soil Health (MISH), which is strongly correlated with conventional soil health metrics and management practices [109]. On a macro scale, locally developed models utilizing soil bacterial genera have been shown to forecast up to 65% of maize yield variation within a specific field and, when validated on a global scale, elucidate up to 37% of vegetation variation, suggesting the presence of conserved microbial signatures pertinent to plant growth [112]. Furthermore, a high degree of microbial diversity, particularly at comprehensive phylogenetic scales, has been associated with increased crop yields and ecosystem multifunctionality, notably under low nutrient availability conditions [122].

These predictive models are instrumental not only in forecasting yield and soil health but also in guiding precision agriculture strategies. By identifying pivotal microbial taxa and functions linked to productivity, agriculturalists and land managers can tailor agricultural strategies such as organic amendments or crop rotations to increase beneficial microbial communities, optimize the utilization of resources, and promote agricultural sustainability [169].

## 7. Challenges and Future Directions for Metagenomics as a Predictive Tool in Agricultural Sustainability

Metagenomics has fundamentally transformed the investigation of soil microbiomes, providing unparalleled insights into microbial diversity and function, which are critical for the maintenance of sustainable agricultural production. Nevertheless, the use of metagenomics as a tool for predicting agricultural sustainability at the field scale presents several substantial challenges and opportunities for advancement.

Technical constraints, such as sequencing depth, database selection or biases, and the reproducibility of outcomes, continue to represent a significant impediment, particularly within intricate and variable soil environments. These limitations can adversely affect the precision and dependability of metagenomic predictions [170].

Similarly, a significant amount of microbial diversity and functional genes remain uncharacterized owing to technical limitations, such as fragmented short‐read sequencing, biases in DNA extraction, and incomplete reference databases. These challenges obstruct thorough functional annotation, resulting in many microbial taxa whose metabolic roles remain unclear [9, 170]. Many existing tools and reference databases have been optimized for human‐associated samples, which results in diminished performance and interpretability within agricultural contexts [171]. Furthermore, the extensive data generated necessitate robust computational resources and standardized workflows to ensure consistent and comparable results across studies [31].

A significant future direction involves the incorporation of multiomics techniques that merge metagenomics with meta‐transcriptomics, metabolomics, proteomics and soil physicochemical data, which is important for comprehensively understanding the microbial community and its functional characterization [102, 103]. Similarly, a meticulously curated database, such as RefSoil, is needed to enhance the evaluation of soil microbiomes [171]. These comprehensive strategies can effectively link microbial community structure to ecosystem processes and agricultural sustainability, thereby offering a more thorough understanding of soil health and resilience [172]. However, harmonizing these diverse datasets presents analytical and technical challenges, requires interdisciplinary collaboration and advanced statistical methodologies [102, 103], and translating laboratory predictions to real‐world and field‐scale applications represents another essential challenge.

Many research findings remain confined to controlled experimental settings. Translation insights into practical, scalable agricultural practices mandate field validation, the establishment of standardized protocols, and the development of accessible decision‐support tools for farmers [11].

However, the integration of machine learning annotation, single‐cell genomics, AI and long‐read sequencing holds significant promise for precision agriculture, facilitating the analysis of complex, multisource data to generate practicable insights. Similarly, challenges related to model interpretability, overfitting, and the requisite domain expertise must be addressed to ensure robust and reliable predictions [173].

## 8. Conclusion

Metagenomic tools have advanced from cataloging microbial diversity to offering predictive insights for sustainable agriculture. Initially, they provided snapshots of the rhizosphere microbiomes, functions and beneficial genes of soil, which are crucial for plant growth and ecosystem resilience [30]. Currently, the focus is on predictive microbiome science, integrating metagenomics with computational analysis, multiomics, and AI to identify beneficial microbial functions such as disease suppression and stress tolerance [172]. Predictive models use metagenomic and environmental data to forecast microbial responses to interventions, aiding in the development of biofertilizers and biopesticides [174].

Metagenomics, employed as a predictive tool, provides significant insights into soil health and microbial diversity, thereby facilitating sustainable agricultural practices that contribute directly to the realization of SDGs 2 (Zero Hunger) and 13 (Climate Action).

Combining metagenomics with remote sensing, IoT sensors, and machine learning further enhances precision agriculture through real‐time monitoring for adaptive management of soil health and crop performance [172]. These advancements promise improved food security and soil health by reducing chemical inputs, increasing yields, and fostering resilient agroecosystems [11]. The shift from descriptive metagenomics to predictive metagenomics is crucial for sustainable, microbiome‐informed agriculture, meeting food demand while protecting the environment.

## Author Contributions

**Oluwaseun Emmanuel Shittu:** data curation, research design, writing of original draft. **Jing Ge:** writing, reviewing, and editing. **Olubukola Oluranti Babalola:** formal analysis, review, editing, conceptualization, project management, and supervision.

## Funding

No funding was received for this manuscript.

## Ethics Statement

The data incorporated in this investigation adhere to the ethical guidelines established by the International Journal of Genomics (Wiley Online Library). This research did not involve animal or human participants, nor did it employ data derived from social media or digital platforms

## Consent

All authors have critically assessed the manuscript and have provided their consent for its publication.

## Conflicts of Interest

The authors have nothing to report.

## Data Availability

Data sharing is not applicable to this article as no datasets were generated or analyzed during the current study.
